# Immunogenicity of a foot-and-mouth disease (FMD) vaccine against serotypes O, A, SAT-2, and Asia-1 in the Middle East and many parts of Africa, Southeast Asia and Europe

**DOI:** 10.1186/s12985-025-02698-7

**Published:** 2025-04-11

**Authors:** Momtaz Wasfy, Abdel Hamid Bazid, Mohamed Nayel, Emad B. Ata, Wael K. Elfeil, Mohamed Attia, Magdy Elsayed

**Affiliations:** 1 Middle East for Vaccines (MEVAC®), 2nd Industrial Area, El-Salhya El-Gedida, 44813 Sharquia Egypt; 2 Department of Medicine and Infectious Diseases, Faculty of Veterinary Medicine, University of Sadat City, Abdel Moneim Riad St, Sadat City, 23897 Menofiya Egypt; 3 Parasitology and Animal Diseases Department, Veterinary Research Institute, National Research Centre, 33 El Bohouth St. (Former Tahrir St.), Dokki, Giza, 12622 Egypt; 4 Faculty of Veterinary Medicine, Suez Canal University, 4.5 Km Ring Road, Ismailia, 41515 Egypt

**Keywords:** FMD vaccine, Antigenic match, Immune response, Topotype, Lineage, Virus neutralization test

## Abstract

Control of foot-and-mouth disease (FMD) is hampered by inadequate biosecurity measures, border transcending serotype strains and unavailability of broad coverage vaccines. In this investigation, six FMD antibody-free calves, aged 1.5–2 years, received a tetravalent, inactivated, aluminum hydroxide gel vaccine (Aphthovac-4) containing 6 PD_50_/dose of certain strains for protection against a wide range of strains in the Middle East, Africa, Southeast Asia, and parts of Europe. The vaccine contained 2 strains of serotype A/Asia (A/Asia (A/Iran-05 and A/Ind/40/2000 G-VII), 2 of serotype O (O/Middle East-South Asia topotype and O/Manisa/TUR/69), and one strain each of serotype SAT-2 (topotype VII) and Asia-1 (Sindh-8). Primary and booster doses were administered 3 weeks apart and sera were collected one week after the booster vaccination, preserved frozen then shipped to The Pirbright Institute, UK, for antibody evaluation by virus neutralization test (VNT) against 22 lineages circulating in the targeted regions. Serum titers against test strains of serotype A were high (range = 355– < 1413 or 2.6– < 3.15 log_10_), and those demonstrating relatively lower values included A/Irn/25/18 (G-VII), A/Irn-05 Far-11, A/Iran05 SIS-13 and SEA-97. Serotype O test strains presented higher titers (≤ 1/1413 or ≤ 3.0 log_10_), but O/Cathay, O/Panasia-2 ant-10 and one O/Ind-2001e lineage exhibited somewhat lower values (range (355–1024 or 2.6–3.01 log_10_). Antibodies against SAT-2 test strains (XIV Topotype) ranged between 128 and 178 (1.9–2.2 log_10_) in 5 animals (5/6, 83%), despite the reported high r1 values. Likewise, Asia-1 strain elicited a similar titer range against IRN/1/2020 in the same 5 animals. The 6th animal generally showed one dilution less. The results portray a dynamic antigenic change between the vaccinal and test strains, underscoring the value of strain matching, use of high payload and incorporation of double antigen lineages within each serotype to broaden coverage in enzootic and epizootic situations.

## Introduction

Foot-and-mouth Disease (FMD) is a severe infection of cloven-hooved animals, with remarkable economic impacts. The disease is enzootic in Egypt, the Middle East, most of Africa and many countries in Asia due to the circulation and incursion of causative viruses through under controlled free borders, deficient biosecurity measures and lack of consistent vaccine programs. FMD is caused by *Aphthoviruses* of the family *Picornaviridae* that demonstrates seven serotypes (O, A, SAT-1, SAT-2, SAT-3, Asia-1 and C), though serotype C is thought to be globally extinct [1]. FMD subtypes have been differentiated into over 65 topotypes, genetic lineages, and strains [1, 2] based on geographic distribution and phylogenetic analyses, making the selection of vaccine strains that antigenically match with those circulating in the field a difficult challenge.

In Egypt, FMD was first reported in 1950 in association with 3 main virus serotypes (O, A, and SAT-2) that showed inconsistent predominance over the previous decades. While serotype O was detected all along, serotype A (genotype IV of East Africa) was recognized irregularly until partially replaced by lineage A Iran-05 in 2006, which is a diverse and persistent strain of topotype ASIA that circulates in the Middle East and South Asian countries through 16 sub lineages [2, 3]. Up to ten sub lineages of Iran-05 showing nucleotide variation of more than 5% in VP1, have been identified from Pakistan, including SIS-10 (2010), SIS-12 (2012–2013), HER-10 (2011–2012), FAR-11 (2011–present), and SIS-13 (2014–present) [3]. Other topotypes of serotype A circulated in different parts of the continent and West Asia and most shared > 70% similarity of nucleotide sequences [4]. Recently, one study from Egypt reported the first detection of A/EURO-SA lineage following the importation of animals from countries in Latin America [5].

Serotype SAT-2/EGY-2012 (topotype VII, lineage 2 reemerged in 2012 after a long period of absence, followed by SAT-2 Libya (topotype VII, lineage 3), which appeared in 2018 [4, 6]. SAT-2 viruses from Bahrain, Egypt, Tanzania, Kenya, Eritrea, Uganda, Cameroon, Nigeria, Sudan and the Palestinian Autonomous Territories matched antigenically with SAT-2 Eritrea 98 vaccine strain by the two-dimensional virus neutralization test, while SAT-2 viruses from Libya did not [7]. Currently, SAT-2 topotype XIV originally detected in Ethiopia is prevalent in Jordan and Iraq, involving strains like SAT2/JOR/11/2023, SAT2/JOR/20/2023, and SAT2/JOR/26/2023 [8].

Because of the diversity of FMD strains, vaccine manufacturers are obliged to keep up with the ever emerging and changing viruses for inclusion in specific, matching and protective vaccines [9]. Gubbins and team from the Pirbright Institute [10] suggested a semi-quantitative procedure for the selection of vaccine seed strains, based on the risk of incursion in an area, prevailing lineages, and immune coverage. However, other authors [9–12] reported that a high potency vaccine may be sufficiently cross-protective against field strains even they had shown a poor match in invitro assays.

It is claimed that at least 80% of the animals in a herd must be immunized to prevent FMDV transmission [13]. However, this goal depends on the size of the farm, density of the susceptible population, and target species [14, 15]. The present study was undertaken to evaluate an FMD vaccine (Aphthovac-4, MEVAC) developed using specific serotypes and topotypes that are currently circulating in Egypt and the Middle East and share antigenic relationships with counterpart strains in many parts of Africa, Southeast Asia and Europe. The vaccine was inoculated into cattle and postvaccination sera were collected after a booster dose and analyzed at the Pirbright Institute. The results of immunogenicity are herewith presented and discussed.

## Materials and methods

### FMD vaccine manufacture (Aphthovac-4)

Two strains of topotype A/Asia (A/Iran-05 and A/Ind/40/2000 G-VII) and two from O/Middle East-South Asia (ME-SA) topotype including O/PanAsia-2 (O/Egy/4/2012) and O/Manisa/TUR/69) were used as vaccine seed viruses. Those ME-SA vaccine viruses have been employed in FMD vaccine manufacturing since 1969 and 2009, respectively. Oher vaccine seed viruses in Aphthovac-4 included one lineage of each of SAT-2 (Sat-2/Egy/2012, G-VII) and Asia-1 (Sindh-8) topotype (Table 1).

**Table 1:** FMDV Serotype strains included in APHTHOVAC 4 (MEVAC), 146S antigen payload and VNT viruses circulating in the MENA- and Southeast Asia regions that were tested against the postvaccination sera at the Pirbright Institute (2023)

Serotype	Topotype	Lineage/ Sub-Lineage	Isolate name	Vaccine 146S Antigen payload	VNT panel Lineage/ Sub-Lineage	Isolate name
A	Asia	Iran-05	A/EGY/1/2012	6 µg	ASIA/Iran-05 ASIA/Iran-05 SIS-10 ASIA/Iran-05 SIS-13 ASIA/Iran-05 FAR-11	A/JOR/3/2006 A/IRN/6/2016 A/IRN/23/2018 A/IRN/18/2021
		G-VII	A/IND/40/2000	4 µg	ASIA/G-VII	A/IRN/25/2018
					ASIA/Sea-97	A/TAI/8/2019
O	ME-SA	PanAsia 2/ ANT 10	O/EGY/4/2012 O/Manisa/TUR/69	6 µg 5 µg	ME-SA/PanAsia-2 Ant 10 ME-SA/PanAsia-2 QOM15 ME-SA/Ind-2001e ME-SA/Ind-2001e ME-SA/Ind-2001e ME-SA/Ind-2001e EA-3 CATHAY SEA/Mya-98	O/IRN/3/2021 O/ISR/1/2021 O/TAI/12/2020 O/JOR/2/2017 O/JOR/10/2021 O/SAU/11/2018 O/TUN/1/2022 O/HKN/4/2018 O/VIT/15/2019 O/VIT/19/2019
SAT2	VII		SAT-2/ EGY/2012	8 µg	XIV	JOR 06/2023 JOR/11/2023 JOR/20/2023 JOR 26/2023 IRQ/2/2022
ASIA-1	Asia	Sindh-8	Asia-1/IND/63/72	10 µg		IRN/1/2020

At the biosafety level 3 laboratories of MEVAC for Vaccines, Egypt, each virus was grown separately in baby hamster kidney suspension cells (BHK-21, strain 21) then aseptically harvested and clarified using 1% chloroform. Titers were in the range of 6.6–7.9 log_10_ TCID_50_/ml (the concentration at which 50% of the infected cells displayed cytopathic effect). Two cycles of treatment with 3 mM binary ethylenimine (BEI) were used to inactivate the virus, and any excess was neutralized with sterile 6 mM sodium thiosulphate.

A sample of each inactivated antigen representing at least 200 doses was inoculated into a bottle of monolayer BHK-21 cell culture and examined over 2–3 days, then the medium was transferred to a fresh cell culture bottle and the original monolayer was replenished with a fresh medium. As an in-process innocuity testing, this process was repeated for 2 weeks to ensure the absence of residual live virus [WOAH guidelines, 2022, 16].

Concentration of the inactivated viruses was achieved through tangential flow filtration (TFF, Consieve® Cobetter, catalog no. UFEFL0300050P), followed by elution with Tris-KCl buffer, pH 7.6. Subsequently, sucrose density gradient ultracentrifugation was performed using Beckman Coulter’s centrifuge, with an SW 55 Ti Swinging-Bucket Rotor (CA 92821 U.S.A) at 50,000 rpm for 35 min at 20 °C. The 146S particle in the resulting concentrates were quantified at an absorbance of 254 nm using ISCO 520C Density Gradient system [WOAH guidelines, 2022, 16].

The vaccine was formulated to contain 4–10 µg of each strain per dose (3 ml/calf) corresponding to 6 PD_50_/dose based on earlier findings [15, 17–21] and MEVAC internal results (unpublished data) (Table 1). Sterility and freedom of the viruses from contaminants was checked by microbiological culture and PCR testing. Aluminum hydroxide gel plus saponin were used as adjuvants.

Vaccine safety was conducted at MEVAC by inoculating two healthy seronegative calves via the subcutaneous route with 6 ml (double the recommended dose) of the vaccine and observing any abnormal local or systemic adverse reactions for 10 days (WOAH) [16].

### Cattle immunization

The vaccine was inoculated into 6 local Balady breed calves aged 0.5–2 years old for immunogenicity testing. They were FMD antibody-free based on VNT, with pre-vaccination sera displaying titers > 1/8 against most of the tested 22 lineages, except against two 2 SAT-2 lineages from Jordan that demonstrated titers below 1/11 in one serum, while 3 other sera showed titers less than 16 against one O lineage and a SAT-2 strain. All animals were regarded as negatives [16] and received the primary vaccine dose (3 ml) plus a booster dose after 3 weeks. Two non-immunized control animals (seronegative for FMD) were included in the study, inoculated with saline and monitored at MEVAC during the study period. Only antisera from the vaccinated calves were forwarded to the Pirbright Institute, UK, for serological testing. Those sera were collected 28 days post vaccination (one week after the booster) and preserved frozen at − 20 °C until shipped.

### Virus neutralization test (VNT)

This gold standard test is customarily performed for evaluating the protective immune response of FMD vaccines in vitro. The test was conducted at the Pirbright Institute based on their internal protocol utilizing the shipped calf sera before and after vaccination. Briefly, 22 FMD topotype strains belonging to serotypes O (n = 10), A (n = 6) and SAT-2 (n = 5), representing regional prevalence in many targeted countries in the Middle East, Africa, Asia and Eurasia [8, 17, 22, 23] were used for evaluating the immunogenicity of Aphthovac-4 (Table 1). Control viruses and sera were prepared for use in the test, then the six calf sera were diluted 1/4 in duplicates (2 wells/dilution) to have 1/8 as the first dilution in sets of 96 well tissue culture plates (50 μl volumes) for reaction with each of the virus lineages tested. Four-fold serial dilutions of sera were made up to 1/1413 and each virus lineage was added to a serum set at a volume of 100 TCID_50_/50 μl and incubated for 1 h at room temperature, then 50 μl of IB-RS-2 cells were added at a concentration of 1 X10^6^ per ml diluent (Eagle`s MEM + HEPES (Sigma M-7278 containing amphotericin B, penicillin 10MU, neomycin 25,000 µg/ml, polymixin B 100,000 U/ml). The plates were placed in the incubator at 37 °C and readings were recorded at the 50% endpoint when the control virus titer had reached a maximum value in a minimum of 48 h.

Antibody titers were expressed as the reciprocal of the highest serum dilution using the Kärber method. A logarithmic base change was conducted to modify the obtained log base 4 values into the more common and readily comprehensible log base 10 by multiplying a mathematical factor of 0.6 to distribute the decimal numbers across the base 10.

## Results

At MEVAC, the vaccine safety experiment confirmed the absence any abnormal local or systemic reactions during 10 days after the double dose vaccination. Also, the non-immunized control animals remained negative for FMD antibodies during the monitoring period (28 days).

**Fig. 1:**
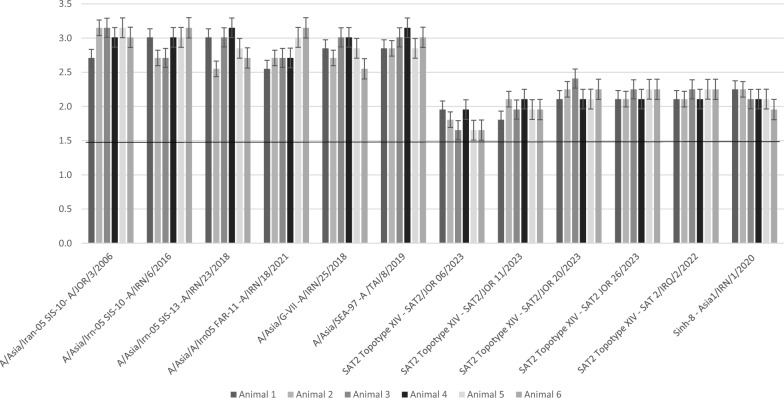
VNT titers Log_10_ against 6 serotype A strains, 5 serotype SAT-2 strains and one Asia-1 strain in 6 cattle receiving Aphtovac-4 (28 days PV)

Results of postvaccination serum titers against 6 FMDV serotype A lineages were generally high against 4 different lineages of A/Irn-05, one A/Asia G-VII lineage and one A/Asia/Sea-97 lineage (A/Tai/8/2019) (Table 1, Fig. 1). Antibody titers against A/Asia/Iran-05 strains (2 SIS-10, SIS-13 and FAR-11) were also high, reaching 1013 (3 log_10_) in 3 of the 4 animals and shot to 1413 (3.15 log_10_) in some of them. Meanwhile, titers against A/Asia G-VII lineage and A/Asia/Sea-97 lineage ranged from 708 to 1413 (2.85–3.15 log_10_).

Despite the presence of one SAT-2 strain belonging to topotype G-II in the vaccine, the sera reacted highly against the tested 5 lineages of topotype XIV, displaying titers between 90 (1.95 log_10_) and 179 (2.25 log_10_), while 2 calves showed relatively lower values against one of the tested strains (SAT-2/Jor/06/2023).

The vaccinal strain of Asia-1 (Asia-1/Ind/63/72, lineage Sindh-08) also produced high antibody titers against a strain from the same lineage (Asia-1/Irn/1/2020) and titers varied between 128 and 178 (2.1–2.2 log_10_) in 5 animals, while the 6th animal demonstrated one log titer less, but above the protection level.

**Fig. 2:**
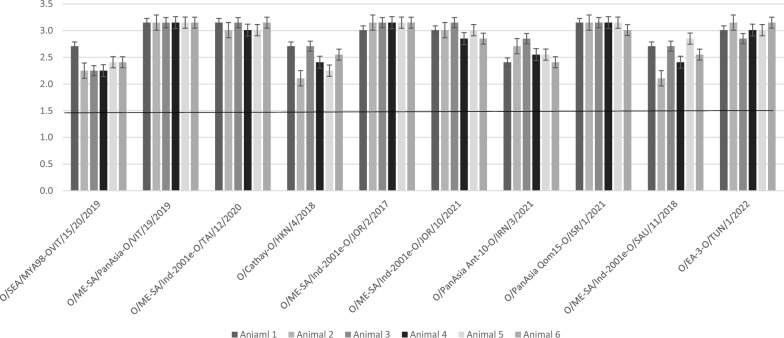
VNT titers Log_10_ against 10 serotype O strains in 6 cattle receiving Aphtovac-4 (28 days PV)

On the other hand, VNT titers against 6 out of the tested 10 serotype O lineages demonstrated high titers up to 1413 (3.15 log_10_), while the remaining 4 lineages O/SEA/Mya98, O/Cathay, ME-SA/PanAsia-2 Ant-10 and ME-SA/Ind-2001e showed relatively lower titers (178–708 or 2.2–2.8 log_10_). Interestingly, while lineage O/Ind-2001e from Saudi was on the lower titer side, similar lineages from Jordan (O/Jor/2/2017 and O/Jor/10/2021) and Taiwan (O/Tai/12/2020 demonstrated higher titers (Fig. 2).

## Discussion

Control of FMD mandates regular vaccination of animals, restriction of movement, slaughter of infected animals and application of hygienic measures. Most enzootic countries are plagued with different serotype strains that spread by animal movement through open or loose boundaries. A polyvalent vaccine may be optimal for controlling the disease and reducing its fulminating impacts, given the diversity of serotypes, topotypes and lineages of FMD viruses that exhibit continuous antigenic and genetic changes. While it is imperative to monitor and characterize the field isolates for a close relationship with the vaccine strain (r_1_-value) [24], the process may not be feasible during outbreaks and emergency conditions.

Since antigen payload is pivotal for stimulating and improving the immune response of an effective vaccine, [25], it is imperative to include adequate antigen quantities per dose based on 146S antigen content. Earlier, tenfold higher antigen doses of a vaccine produced fourfold titer increases against 10 heterologous strains in cattle [15, 25]. However, it should be borne in mind that antigen payloads may be subject to serotype differences and could be influenced by antigen stability and type of adjuvant used [26]. Accordingly, vaccines were required to pass serological testing for effectiveness and potency evaluation before release [26].

The potency test [27] is performed when matching of the vaccinal strain is suspected against the field strain. Here, animal groups are inoculated with different amounts of the vaccine and resistance to live virus challenge is monitored to calculate the number of protective doses contained. Generally, a vaccine dose that protects 50% of the vaccinated animals against clinical disease after challenge is referred to as having one PD_50_. The WOAH minimum standard for FMD vaccines is 3 PD_50_ per dose in enzootic settings [26], while those with > 6 PD_50_ per cattle dose were claimed to provide a broader range of immunity and protection against homologous and many heterologous challenge viruses, even those with low r-values (11, 26, 27, 28]. However, the method of potency testing suffers from low precision, potential biological variation in the field, invasiveness, inconsistency, environmental risk and high cost [15, 28, 29].

Many studies have analyzed the correlation between the antigen payload and PD_50_. Concentrations of 146S antigen ranging between 1.5 and 9.2 µg/dose were recommended for protective vaccines [18, 19]. In bivalent O/A vaccines, Li et al*.* [20] found that 146S concentration between 4.72 and 38.90 µg/dose corresponded to PD_50_ values from 7.05 to 15.59. For SAT-1, SAT-3 and SAT-2 strains, 3.0 µg/ml, 3.0 µg/ml and 6.0 µg/ml resulted in 4 and 32 PD_50_, while, one SAT-2 vaccine at 6.0 µg/ml showed 4–10 PD_50_, with an antibody titer ≥ 2.0 log_10_ from day 7 PV [21]. These reports are in agreement with the internal findings of MEVAC [unpublished data], suggesting the use of a range of 4–10 µg of each vaccinal strain to constitute 6 PD_50_/dose per dose (Table) [14, 20, 21]. Aluminum hydroxide-saponin was used as an adjuvant for performing at least as effectively as oil adjuvants [15], with the advantage of a lower price.

As far as serotype A antigens in Aphthovac-4 are concerned, A/Iran-05 and A/IND/40/2000 (G VII) lineages were included in the vaccine, since A/IRN-05 was antigenically related to sub lineages belonging to those circulating in the Middle East and neighboring countries, like Iraq Turkey, Afghanistan and Pakistan (A/PAK/30/2016, PAK/73/2019) [27, 30]. It also barely matched with A/Tai/17/2019 and A/Tai/8/2019 [31]. On the other hand, the vaccinal strain A/IND 40/2000 (G-VII) has originally emerged in the Indian sub-continent during 2003 then moved by 2015 to Iran, the Middle East, Saudi Arabia, Turkey and West Eurasia [1], although its antigenic relationship with others is not well understood [1, 32]. The present study may be the first to use this combination of vaccinal strains to evaluate their combined immunogenicity against a number of lineages known to be antigenically heterologous, including A/IRN-05 SIS-10, A/IRN-05 SIS-13, A/IRN-05 FAR-11, A/IRN-05 G-VII of Saudi Arabia [9], not to mention A/Asia/Sea-97 strain.

Results of VNT against the 6 A/Asia topotype A lineages chosen from diverse geographical locations, with potentially dissimilar antigenic composition showed a strong antibody response, most probably in association with the high PD_50_/dose (Fig. 1) and the presence of both A/Iran-5 lineage, and A/IND/40/2000 (lineage G-VII) in Aphthovac-4 (Table 1, Fig. 1). Overall, the titers ranged between 355 (2.6 log_10_) up to 10 1413 (3.15 log_10_), with an average of 877.4 + 337.0 (2.9 log_10_). Titers against A/Asia/Iran-05 strains (2 SIS-10, SIS-13 and FAR-11) reached 1013 (3 log_10_) in at least 3 of the 4 animals tested against these lineages and shot to 1413 (3.15 log_10_) in some of them. Only one calf demonstrated a relative decrease in titer against A/Irn-05 Far-11 and another showed a similar decrease with A/Iran05 SIS-13, probably due to their own immune mechanisms [17].

Antibody Titers against A/Asia G-VII lineage and A/Asia/Sea-97 lineage were also high ranging from 708- 1413 (2.85–3.15 log_10_). Overall, all titers recorded in this study were much higher than the recommended protection threshold (/32–1/45), despite the potential antigenic or genetic differences that may affect the reactions between the vaccinal and test lineages [2, 33–35]. For instance, one study reported that a combined vaccine containing both A/IRN/05 and A/SAU/95 induced protection in 56% of the vaccinated animals after challenge with A/Asia/G-VII virus, while a vaccine from A Malaysia 97 (A/ASIA/Sea-97) at > 6 PD_50_ was protective against the same heterologous challenge virus [28, 35].

The panel of O serotype lineages used for VNT testing involved ten currently circulating lineages in countries of the Middle East, Africa, Southeast Asia and Eurasia (8, 17, 22, 23]. Generally, postvaccination sera demonstrated high titers in association with the vaccinal combination used, high payload and good matching profiles (1413 = log_10 _> 3.15, Fig. 2). Out of the tested 10 serotype O lineages, 6 had titers up to 1413 (3.15 log_10_), while the remaining 4 lineages O/SEA/Mya98, O/Cathay, ME-SA/PanAsia-2 Ant-10 and ME-SA/Ind-2001e showed values between 256 (2.4 log_10_) and 512 (2.7 log_10_). These results are supported by findings from the reference laboratories, indicating a broad match between the vaccinal strain O/Manisa and EA-3 strains from Algeria, Ethiopia, Sudan, Israel, and Pakistan,as well as O/Camos, O/Tur/15/2009, and O/3039 [35, 36]. In addition, the vaccinal strain O/PanAsia-2 showed a good match of more than 80% with O serotype isolates in SEA, East Asia and the Far East, same as other regional vaccines containing O/Cathay (O/HKN/6/83), O/SKR/2010 and O/MYA/2009 [37]. O/PanAsia-2 also matched with O-Ind-2001d strains, which emerged in the Indian subcontinent around 2008, and spread to Vietnam, Laos and Myanmar in 2015–2016 [37, 38]. Shortly, sub lineages of this strain (O/ME-SA/Ind-2001d and O/ME-SA/Ind-2001e) appeared in North Africa, the Middle East, and East Asia [37].

Antibody titers against 4 lineages (O/Cathay, O/SEA/Mya-98, O/PanAsia-2 Ant-10 and one strain of O/Ind-2001e) were somewhat lower than others (range = 178–708 or 2.25–2.85 log_10_, averaging = 350.5 ± 172.4 or 2.5 log_10_). Strain O/Cathay (Archaic for China) has been implicated in severe porcine outbreaks in China and Southeast Asia, including Hong Kong, Taiwan and Vietnam. Virulence and ability of this topotype to spread to cattle was reportedly lower than that in pigs [39] due to its peculiar antigenic structure and several capsid amino acid substitutions at the neutralizing antigenic sites [39]. Although a vaccine from O/Cathay O/HKN/6/83 was shown to be protective in an earlier study [39], recent findings indicated that none of the recently circulating Cathay viruses were covered by this vaccine because of an antigenic drift [40].

The decrease in titers observed with the other 3 lineages (range 178–708 or 2.2–2.8 log_10_) may probably reflect amino acid substitutions and antigenic differences between the vaccinal and test strains, though all values were much higher than the recommended protection threshold [41]. Interestingly, while the average titer of strain O/Sau/11/2018, being among those displaying a lower titer (O/Ind-2001e, 411.8 ± 207.8), others strains belonging to the same lineage (O/Tai/12/2020, O/Jor/2/2017 and O/Jor/10/2021) induced titers up to 1413, probably suggesting less antigenic differences, not to mention the presence of the combined antigens in the vaccine and the high payload [11, 15]. The findings are in consent with the previous notion that heterologous response to a vaccine containing two FMDV strains was superior to that obtained from one strain only [25]. For instance, O/Manisa marginally matched with O Ind/2001d (r1 < 0.3), but a vaccine from it in addition to the moderate match strain O3039 at > 6 PD_50_/dose elicited protection against O/ALG/3/2014 (O Ind/2001d) [41]. It is noteworthy that both O/Manisa and O/ME-SA strains in the vaccine exhibited a high antigenic match with topotypes O EA-3 and O EA-4 in East Africa, supporting a broad immunogenic coverage in this region [42, 43]

The third antigenic component of the vaccine, serotype SAT-2, topotype G VII (SAT2/ EGY/A/2012) was included due to its prevalence in Egypt following earlier incursion by unobserved carriers from Sudan, Nigeria, Saudi Arabia, Libya, Eritrea, and Cameroon during outbreaks [20, 44]. This topotype has been claimed to antigenically match with the currently circulating topotype XIV strains in Bahrain (SAT2/BAR/2/2022 and SAT2/BAR/7/2022), Ethiopia (SAT2/ETH/2/2022, SAT2/ETH/3/2022) and Iraq (SAT2/IRQ/2/2022, SAT2/IRQ/5/2023 and SAT2/IRQ/9/2023) [9]. However, SAT-2 VIX witnessed multiple introductions into the Middle East, with the Jordanian sequences not interleaved with those from Iraq [8]. Similar findings were also reported for SAT-2 strains in Tanzania and the Palestinian Autonomous Territories [44]. Interestingly, the latest SAT-2 viruses from Libya did not match with either SAT-2 Eritrea or SAT-2 Zim vaccine strains [44].

In the present study, VNT was conducted against 5 viruses belonging to SAT2 XIV topotype strains circulating in Africa, Jordan and the "Kurdish triangle". The obtained results support previous reports of high cross-reactions between SAT-2 topotypes VII and XIV in 4/6 animals (83%) [4, 8], with a mean titer of 138.3 ± 34.0 (range = 128–178 or 2.1–2.25 log_10_), all above the protective threshold (Fig. 1). The other two animals showed somewhat lower titers against this lineage, but all differences were in line with the presumed 0.4 log_10_ standard deviation differences in VNT results per se or the peculiarities of the innate immune response of individual animals [17].

The observed decrease in SAT-2 VII titers against G-XIV virus may be duly attributed to antigenic differences between the vaccinal and test topotype lineages [7] together with the inherent low thermostability of SAT-2 antigens, which affects the structural integrity of virions and reduces the immune response, even after several vaccinations [44]. Differences in stability due to pH and storage buffers have been reported between and within SAT-2 topotypes, resulting in vaccines with low protective capacity [45]. Approaches to improve capsid stability have been proposed, including the use of chimeric strains or mutant strains in vaccine manufacture [20]. Some workers suggested the addition of chemical stabilizers (a combination of trehalose, 500 mM NaCl and 3 mM CuSO_4_·5H_2_O) before BEI inactivation [20] to enhance the thermostability. However, all of these methods need to be validated and proven in the field at production level. Overall, the present study may be the first to demonstrate the possibility of using SAT-2 G VII vaccine to immunize against G XIV topotype strains that are circulating in many surrounding countries in Africa and Asia [4, 8].

The fourth antigenic component of the vaccine is Asia-1/IND/63/72 (lineage Sindh-8), was tested against a strain of the same lineage (Asia-1/IRN/1/2020). It showed high titers in 5 animals (mean = 138.3 ± 34.1, Fig. 1), but relatively less in the 6th. This may be due to animal-to-animal variation in immune response or the presence of antigenic differences between the vaccine and test virus as part of the ongoing temporal and topographic antigenic variations within FMDV serotype strains. Phylogenetic analysis of VP1 gene sequences showed Asia-1 virus to consist of 7 lineages, Sindh 08, G-I, G-VIII, G-Vib, G-V, G-IX and G-III [46]. Initially, all were thought to be cross protective and WOAH has recommended Asia-1 Shamir strain for vaccine production [47], but the need for using different or additional lineages for vaccine manufacture deemed inevitable in response to differences in antigenic matching and protection results [47]. Asia-1 lineages are enzootic in southern Asia (Afghanistan, India, Iran, Nepal, Pakistan), north Asia (Kyrgyzstan, Tajikistan, Uzbekistan), several regions in China, Mongolia, Eastern Russia, and North Korea.

Conclusively, it appears that FMD vaccination with Aphthovac-4 has resulted in protective antibody responses against all tested lineages and sub lineages from different countries in the Middle East, and parts of Africa, Asia and Europe. This is of value in enzootic situations and at emergency conditions, supported by the high antigen payload and combination effect of lineages that cross-reactive with many field challenge strains. Relative differences in antibody titer levels against some of the test strains were associated with antigenic differences that need to be monitored and delineated. The current study may be the first to use a combination of vaccinal strains, like A/Iran-05 and A/IND/40/2000 (G VII) against a number of lineages known to be antigenically heterologous. The study also tried employing SAT-2 G VII topotype antigens for protection against topotype SAT-2 G XIV strains, confirming the utility of such vaccines in controlling FMD.

## Data Availability

No datasets were generated or analysed during the current study.
